# Early different cognitive processes evoked by carnival vs. general promotions when shopping online: An ERPs study

**DOI:** 10.3389/fnins.2022.938511

**Published:** 2023-01-09

**Authors:** Wei Han, Xuefeng Zhang

**Affiliations:** School of Management, Southwest University of Political Science and Law, Chongqing, China

**Keywords:** carnival promotion, general promotion, cognitive process, P2, N2

## Abstract

**Introduction:**

The booming development of online shopping has intensified market competition. In addition to general sales promotions, online shopping has introduced new changes including artificial carnival promotions.

**Method:**

This study aims to investigate cognitive processes to an unknown e-commerce platform after exposure to carnival and general promotion activities using event-related potentials. Thirty-three participants were recruited in this study to probe how consumers perceive carnival and general promotion information using event-related potentials (ERPs). Carnival or general promotion posters were presented first, then an unknown e-commerce platform brand was presented in the second stage, at which time the subjects’ cognitive process to the brand were observed in an implicit paradigm.

**Results:**

The results showed that after priming with carnival promotion posters, the unknown e-commerce platform stimuli elicited larger P2 and N2 components than were observed after the presentation of general promotion posters; however, the P3 component did not show a significant difference. These findings indicate that the target identification and cognitive control mechanism with regard to an unknown e-commerce platform are likely influenced by the implicit memory of different promotion activities when shopping online.

**Discussion:**

The results suggest that ERP components may have the potential to be employed as indices to estimate the effectiveness of promotion methods for an unknown brand.

## 1. Introduction

Intense competition in the field of online shopping has triggered multiple promotion approaches in order to capture market share and achieve business goals (Dhruv et al., 2010). General promotion approaches, such as cash discounts, coupons, and other conventional means of promotion (Peng et al., 2019; Sinha and Verma, 2020; Kotler et al., 2021), can be frequently observed throughout the year. However, these promotion approaches have become so ordinary that they lack attraction for consumers, who have gradually become familiar with the basic marketing logic of these methods (Amornpetchkul et al., 2018; Pan et al., 2022). It is difficult to say whether a wide range of general promotion activities will be successful; even if so, they may require a huge advertising budget. In this context, online carnival promotion, “which is featured with playful spirit and festival atmosphere, is becoming a key strategy to engage customers and boost sales for e-commerce platforms” (Shi et al., 2022), has drawn much attention from scholars and practitioners (O’Sullivan, 2016; Zhao et al., 2019).

As a new type of event marketing and holiday promotion, online shopping carnivals are regarded to be the most successful type of promotion campaign. Characteristic examples are the *DOUBLE ELEVEN* online shopping carnival held since 2009 by Taobao&Tmall^®^ in China or *Cyber Monday* in the United States (Swilley and Goldsmith, 2013; Liu et al., 2021). It has been shown that an artificial online shopping carnival can stimulate consumption by offering pronounced discounts and by creating a festive atmosphere (Yu et al., 2018; Xu et al., 2020). During the particular online shopping carnival period, the number of participating brands and consumers will substantially increase, and the transaction amounts will also increase steeply. For example, the sales volume during the *DOUBLE ELEVEN* carnival increased from 50 million RMB in 2009 to 540.3 billion RMB in 2021, representing a more than a 10,000-fold increase in 10 years. Compared with Taobao&Tmall^®^’s average daily sales in 2021 (19.7 billion RMB), the sales of the *DOUBLE ELEVEN* day reached near-mythical proportions.

There are at least two significant differences between carnival promotions and general promotions. First, unlike general promotions that mainly focus on price discounts, carnival promotions emphasize the frenzied shopping atmosphere that has been fostered, which can be described as a “second life” to consumers that is opposite to consumers’ structured lives (O’Sullivan, 2016; Xu et al., 2017). Just as O’Sullivan (2016) argued that “eccentricity, profanities, suspension of hierarchies and emotion bond” are the four features of “carnival,” consumers often enjoy playful carnival promotion activities, such as shopping games, gifts, and shows, and share their pleasure with others. Second, the trading volume and transaction amounts will be considerably enlarged (Yu et al., 2018) due to the dramatically increased number of participating merchants (Liao et al., 2022) and much bigger discount magnitude than during general promotions, even reaching half-price or less (Xu et al., 2015), as well as the overwhelming increase in advertising on television, the internet, social media networks, and newspapers. Furthermore, the number of participating consumers will increase dramatically since herd behavior is evoked (Liao et al., 2022).

Given the differences between carnival promotions and general promotions, we argue that consumers may have different cognitions when confronted with the two promotional approaches. First, when encountering a carnival promotion, consumers might intend to get involved in the “second life” to enjoy the frenzied atmosphere and emotional bonds with others (Yan et al., 2016). This herd mentality may trigger consumers’ positive attitudes during or even before the carnival promotion campaign. Second, since the extent of the price discounts during a carnival promotion is normally higher than during a general promotion (Kim and Krishnan, 2019), consumers might take carnival promotion activities as an opportunity to store goods and save money at the same time (Jee, 2021). Accordingly, we believe that although carnival promotions may have a dark side such as low delivery efficiency (Ma et al., 2022), they still attract more attention from consumers than do general promotions.

Researchers have attached great importance to online shopping promotion activities in marketing research, and existing studies have mainly focused on the following aspects. The first of these is the development process of online shopping promotion and the situation of consumer adaptation (O’Sullivan, 2016), such as combining online shopping with traditional festivals (Swilley and Goldsmith, 2013) or artificially creating a custom shopping festival such as the *DOUBLE ELEVEN* (Li et al., 2022). Second, they have examined the influence of online shopping promotions, especially carnival activities like *DOUBLE ELEVEN*, on the enterprises that initiated the promotion activities, such as the enterprises and the consumers who participated in the promotion activities (Pan et al., 2022), the enterprises’ sales performance (Svatosova, 2020), the number of people participating, and the changes in consumers’ shopping habits (Amornpetchkul et al., 2018). Third, they have examined the influence of the form, intensity, and duration of online shopping promotion activities on consumer attitudes (Jee, 2021), consumer purchasing intention, and consumer purchasing decision (Chen and Li, 2020). Fourth, they have examined the essential attributes of online promotion activities, such as the differences between online shopping promotions and traditional retail promotions in terms of time (Qihua et al., 2020; Akram et al., 2021), space, enterprise, atmosphere created by the promotion, and consumer choice (Chen and Li, 2019; Schneider and Zielke, 2019). They have also explored other research topics related to promotional activities, such as logistics services and financial services that support online shopping promotions (Wei et al., 2016; Qadan et al., 2020). However, the studies conducted on the topic of online shopping carnival promotions have mostly focused on established retailing platforms (Xu et al., 2017; Li et al., 2022), with less concern for unknown platforms. Taking an established retailing platform as the research context implies that the effect of a carnival promotion on consumers’ purchasing behavior is mixed with the reputation of the established platform. Undeniably, an established well-known platform plays an essential role in the endorsement of a carnival promotion (Chen and Li, 2020; Xu et al., 2020). Thus, we cannot clarify the independent effect of carnival promotions on consumers’ behavior, which means that less is known about whether lesser-known enterprises could replicate such success through carnival-style promotions. Accordingly, it is interesting to explore whether online carnival promotions could have a similar impact on consumers if the online carnival promotion were held by an unknown platform.

To test that question, we used a reliable physiological experimental method, event-related potentials (ERPs), to conduct this research. Prior relevant research has normally adopted questionnaires (Faical et al., 2015), interviews (William and Timothy, 2012), or market surveys (Dhruv et al., 2011) as research methods. Because of data collection bias (Baldo et al., 2022; McInnes et al., 2022; Oliveira et al., 2022), these methods are not helpful in probing consumers’ cognitive processes. With the help of new neuroscience techniques, such as functional magnetic resonance imaging (fMRI), ERPs, or eye-tracking, the processes by which information is received and processed can be observed. Neuroscience can provide a novel way to establish links between cognitive processes and traditional marketing data (Karmarkar et al., 2021). Use of these neuroimaging tools is a more reliable and robust way to test the relationship between online shopping promotion methods and consumer cognition at a higher level of accuracy than is possible with current marketing tools (Zhang, 2020). Furthermore, carrying out such research can provide a more comprehensive assessment of the efficacy of marketing techniques, such as advertising, consumer competitions, and product placement, by analyzing the underlying neurobiology (Baldo et al., 2022).

Using ERPs, this study attempts to investigate differences in consumers’ cognition when processing two different online promotion activities in the context of an unknown retail platform in a laboratory setting, specifically general promotion and carnival promotion. Scholars have pointed out that ERP components N1 and P2 may reflect the orienting of attention toward task-relevant target stimuli (Fuggetta and Duke, 2017; Hong et al., 2020). N2 may reflect the process of cognitive control (Chaillou et al., 2018; Zabelina and Ganis, 2018) or the modulation of the detection of novel stimuli and the orienting of visual attention (Estate et al., 2009; Hoffmeister et al., 2022), while ERP components P3 may reflect the categorization (Dieciuc et al., 2017) or the allocation of attention (Kranczioch and Dhinakaran, 2013; Harper et al., 2019). Based on the cognitive processes reflected by the above ERP components and on the results of similar prior ERP studies, we argue that consumers’ cognitive process toward an unknown retailing platform could show significant differences after exposure to the two different online promotion activities mentioned above. Specifically, we expect that subjects’ orienting of attention, cognitive control process, and categorization process toward the unknown e-commerce platform may show differences that are intrinsically due to the different promotions and that this could be reflected by the components N1, N2, P2, or P3.

Our research attempts to make several important contributions to the existing literature. First, our research may provide some evidence that there exists a positive link between carnival promotion and consumer cognition in the context of an unknown retailing platform, expanding the applicable scope to unknown platforms or brands by eliminating the effect of a well-known platform’s reputation. Second, our work may enrich the literature on online carnival promotion by clarifying the effect of carnival promotion on consumer cognition rather than simply behavior. Third, our work may provide a more comprehensive assessment of the efficacy of online carnival promotion, by analyzing the underlying neurobiology and determining which ERP components may serve as sensitive indicators to promotion approaches.

## 2. Materials and methods

### 2.1. Subjects

A total of 33 college students (16 men and 17 women; mean age 23.4 ± 2.7 years) were recruited. All participants reported normal or corrected-to-normal vision. None of them had any history of neurological or psychiatric illness, head trauma, or drug abuse, and none were taking medication. All participants were right-handed and native Chinese speakers. Each subject completed written informed consent forms from the local medical ethics committee, in accordance with the Declaration of Helsinki. Each participant received a small gift worth about 5 USD as compensation for their participation.

### 2.2. Stimulus materials

**FIGURE 1:**
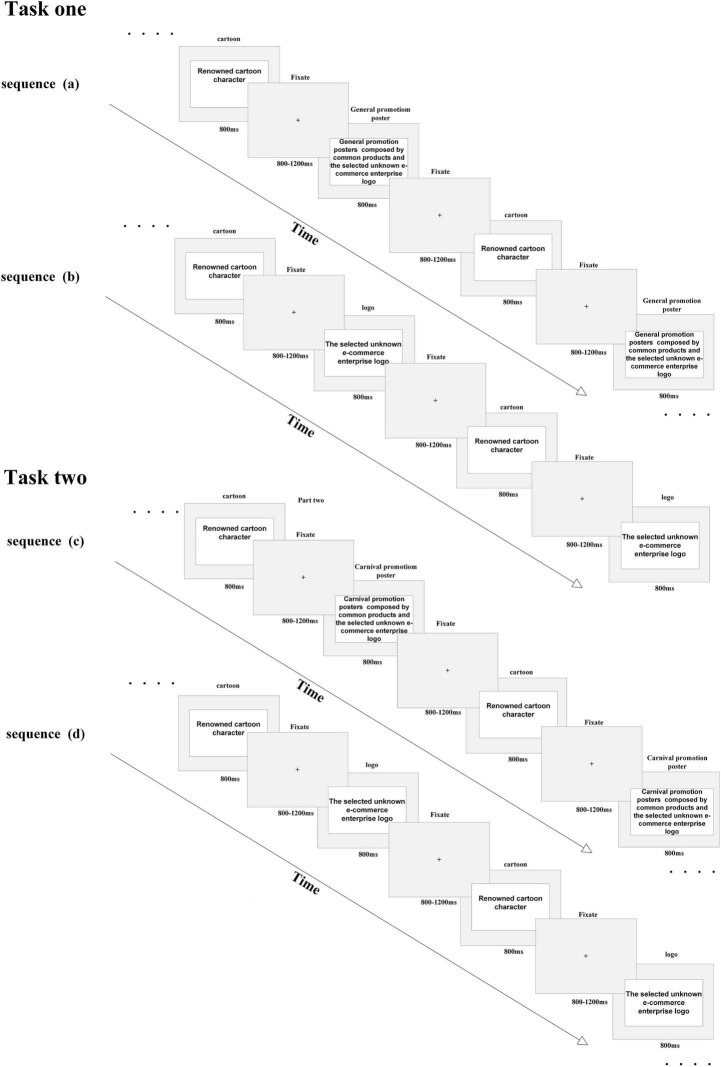
Schematic drawing of the paradigm. Task 1 contains sequence A and sequence B. Sequence A was composed by cartoon pictures and general promotion posters with brand in an amended oddball paradigm. Sequence B was composed by cartoon pictures and e-commerce logo in an amended oddball paradigm. Task 2 contains sequence C and sequence D. Sequence C was composed by cartoon pictures and carnival promotion posters with brand i n an amended oddball paradigm. Sequence D was composed by cartoon pictures and e-commerce logo in an amended oddball paradigm. Subjects’ task was to identify and verbally report the number of cartoon character to researcher at the end of every sequence. Discount forms and discount magnitude, cash discount, discount coupon, time-limited sales promotions, and quantity discount used in general and carnival promotion posters, other promotion methods such as coupons, free gifts, and lottery were also used in posters. Overall, the promotion magnitude in carnival promotion posters was greater than in general promotion posters.

The primary classes of stimuli and the timing of the experiment are shown in Figure 1. The critical stimuli were three colorful cartoon character pictures, five online shopping platform brand logo pictures with different styles, five general promotion posters that included the online shopping platform brand logo, and five carnival promotion posters that included the online shopping platform brand logo. The cartoon characters were all familiar to the subjects. An unknown but real online shopping platform was chosen for this study to eliminate the influence of well-known online shopping platforms’ reputations. Compared with the other online shopping platforms’ market shares, such as the top-ranked Tmall at 55.7% and second-ranked Jingdong Mall at 25.1%, the selected platform’s market share was less than 0.5% according to China’s e-commerce market data monitoring report in 2019, published by the China e-Business Research Center^®^. The products presented in both the general and carnival posters were selected from four categories, namely, daily necessities, drinks, foods, and electronic goods. These categories were chosen because they were closely related to the subjects’ everyday lives. The debriefing verified that the subjects were familiar with the selected products presented in the posters, but that they had not heard of the e-commerce platform before, nor had they shopped from it.

Following the example of prior studies (Yu et al., 2018; Liao et al., 2022) and after the observation of the magnitudes of carnival promotions like *DOUBLE ELEVEN* or *Cyber Monday*, two sets of paired promotion posters were designed, and the promotion reasons varied slightly depending on the promotion’s content. Many differences between carnival and general promotions, such as discount forms and discount magnitude, cash discounts, discount coupons, time-limited sales promotions, and quantity discounts, were used when designing the posters to reduce interfering options, again following the example of previous studies (Kumar et al., 2004; McKechnie et al., 2012). The promotion time was set to end within a week after the experiment, to better attract subjects’ attention.

### 2.3. Experimental procedure

The experiment consisted of two tasks, as shown in Figure 1. Task one comprised sequence A and sequence B, and task two comprised sequence C and sequence D. Sequence A consisted of three cartoon pictures and five general promotion posters. Sequences B and D consisted of three cartoon pictures and three online shopping platform brand logo pictures. Sequence C consisted of three cartoon pictures and five carnival promotion posters. The e-commerce platform brand logo was visible at a fixed position (upper right corner) in sequences A and C. An amended oddball task was used in this study: the cartoon character pictures were taken as target stimuli in sequences A and C; promotion poster pictures or online shopping brand logo pictures were taken as standard stimuli in sequences B and D. The experiment was divided into two parts to minimize the potential carry-over effects of the experimental sequence. Subjects first performed task one, which consisted of sequence A followed by sequence B after a break. Two weeks later, subjects performed task two that consisted of sequences C and D separately by a break.

The experiment was carried out in an electrically shielded and sound-attenuated experimental chamber. Subjects sat in a comfortable chair while performing the task, which was programmed in, and presented by, E-prime professional (vision 2.0, Psychology Software Tools, Sharpsburg, MD, USA). The stimulus pictures in each sequence were presented in a pseudo-random order, in which every stimulus was presented 30 times. Each trial in the experiment began with a screen-centered fixation cross (random inter-trial interval with a duration between 800 and 1,200 ms) presented in light gray against a black LCD computer screen, which was then replaced by the presentation of a stimulus for a duration of 800 ms. All stimuli subtended a horizontal visual angle of 10.3° and a vertical visual angle of 6.8° at a viewing distance of 1 m between the subject and the screen center. The subjects’ tasks were to identify and verbally report the number of presented cartoon pictures at the end of every sequence. To ensure the validity of the experiment, if the accuracy was less than 95%, the data would be discarded. Before the official experimental blocks, subjects performed one training block to familiarize them with the task.

### 2.4. Electroencephalogram recording and analysis

While performing the experimental tasks, subjects wore a 32-channeled electroencephalogram (EEG) cap (Quick-Cap, Neuroscan, VIC, Australia), with electrodes placed according to the International 10/20 system. A reference electrode was placed on the left mastoid, and a ground electrode was placed on the midpoint of FpZ and Fz. Vertical and horizontal electrooculograms (EOG) were monitored. Impedances for all electrodes remained below 5 KΩ during the entire acquisition process. The EEGs were recorded using the Neuroscan^®^ EEG system (Neurosoft Labs Inc., VIC, Australia) as the subjects performed sequences B and D. The acquisition process was continuously recorded with a bandpass of 0.01–100 Hz and a sample rate of 500 Hz. Offline data were processed using Curry7.0 SBA (Neurosoft Labs Inc.). Serious artifacts caused by eye and other muscular movements were removed manually. Trials in which the base-to-peak EOG amplitude exceeded 200 μV, amplifier saturation occurred, or the baseline shift exceeded 250 μV/s were automatically rejected offline, with the result that a total of 6% of the data were rejected. ERPs were segmented into time-locked epochs using the onset of the e-commerce platform logo pictures as a reference, starting 200 ms before to 800 ms after the presentation. The mean amplitudes of ERP components evoked by the unknown online shopping brand logo were computed based on the EEG elicited in sequences B and D after different promotions using within-subject repeated-measures ANOVA.

## 3. Results

**FIGURE 2:**
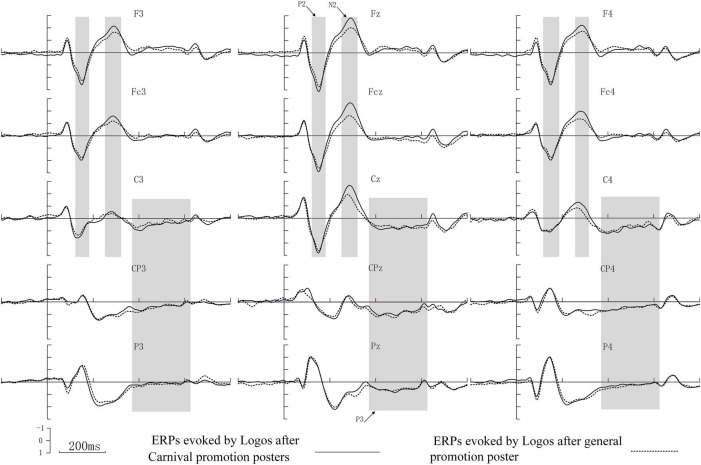
Raw ERP waveforms at 15 electrode sites. Grand averaged ERP elicited by logos after carnival promotion posters (solid line) vs. logos after general promotion poster (dotted line) at 15 electrodes in the frontal, central, and parietal areas. Arrows indicate the P2 around 120–180 ms, N2 around 270–320 ms, and P3 around 370–630 ms. The ERP components P2 and N2 evoked by logo after carnival posters was stronger than after general promotion posters; however, the ERP component P3 evoked by logo after two promotion posters did not show significant differences.

**FIGURE 3:**
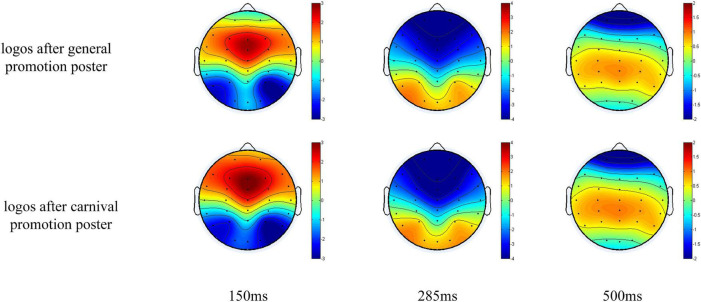
Topographic maps of the voltage field topography at the peak of the P2, N2, and P3 evoked by logos after general and carnival promotion poster. Red and yellow are positive, blue and black negative, scaled from –3 to 3 mV (P1), –4 to 4 mV (N2), and –2 to 2 mV (P3).

The ERP components evoked by the e-commerce platform brand logo in the frontal, central, and parietal areas are presented in Figure 2. Fifteen electrodes (F3, Fz, F4, FC3, FCz, FC4, C3, Cz, C4, CP3, CPZ, CP4, P3, Pz, and P4) were selected for statistical analysis based on a visual examination of the potential distributions and topographical maps of the scalp (Figure 3), as well as best practices from the previous literature (Geoffrey et al., 1996; Anika et al., 2013). A within-subjects measures ANOVA was conducted on the mean amplitudes of P2, N2, and P3. The mean amplitudes of the frontal-central P2 (120–180 ms) and N2 (270–320 ms) were analyzed to examine neural responses to the unknown e-commerce platform brand logo after the presentation of different promotions. Nine electrode sites (F3, Fz, F4, FC3, FCz, FC4, C3, Cz, and C4) were chosen for the analysis of P2 and N2. ANOVA factors were stimulus type (two statuses: after general promotion vs. after carnival promotion) and electrodes (nine sites: F3, Fz, F4, FC3, FCz, FC4, C3, Cz, and C4). Similarly, nine electrode sites (C3, Cz, C4, CP3, CPZ, CP4, P3, Pz, and P4) were chosen for the analysis of P3 (370–630 ms) in ANOVA. As appropriate, the Greenhouse–Geisser correction for degrees of freedom and contrast analysis was used. The significance level was set to a *p*-value of <0.05. The basic descriptive statistics of each component evoked by different stimuli at selected electrodes are listed in Table 1.

**TABLE 1:** Basic descriptive statistics of evoked potentials.

Distribution		After general promotion	After carnival promotion	*t*-Value	*P*-value
F3	P2	1.79 ± 0.93	2.09 ± 1.09	-2.296	0.028
	N2	-2.38 ± 0.93	-3.09 ± 1.41	2.295	0.028
Fz	P2	2.05 ± 1.00	2.43 ± 1.15	-2.775	0.009
	N2	-2.95 ± 0.75	-3.64 ± 1.39	2.665	0.012
F4	P2	1.38 ± 0.96	1.86 ± 0.97	-4.525	0.000
	N2	-2.57 ± 0.85	-3.28 ± 1.40	2.660	0.012
FC3	P2	1.63 ± 0.50	1.79 ± 0.56	-2.208	0.035
	N2	-1.70 ± 0.63	-2.32 ± 1.11	2.422	0.021
FCz	P2	2.07 ± 0.66	2.33 ± 0.89	-2.240	0.032
	N2	-2.52 ± 0.49	-3.11 ± 1.12	2.693	0.011
FC4	P2	1.15 ± 0.60	1.43 ± 0.79	-3.045	0.005
	N2	-1.76 ± 0.60	-2.33 ± 0.98	2.712	0.011
C3	P2	0.99 ± 0.29	0.73 ± 0.43	3.465	0.002
	N2	-0.58 ± 0.39	-0.83 ± 0.59	2.994	0.005
Cz	P2	1.44 ± 0.48	1.73 ± 0.63	-3.151	0.004
	N2	-1.67 ± 0.47	-2.24 ± 0.78	3.498	0.001
C4	P2	0.14 ± 0.48	0.36 ± 0.73	-2.601	0.014
	N2	-0.33 ± 0.76	-0.92 ± 0.91	4.012	0.000
C3	P3	0.47 ± 0.25	0.22 ± 0.26	2.651	0.012
Cz	P3	0.59 ± 0.29	0.57 ± 0.34	1.561	0.013
C4	P3	0.21 ± 0.21	0.38 ± 0.23	-0.805	0.027
CP3	P3	0.73 ± 0.40	0.55 ± 0.41	-0.911	0.036
CPz	P3	0.36 ± 0.27	0.33 ± 0.45	1.681	0.012
CP4	P3	0.63 ± 0.69	0.14 ± 0.76	-0.654	0.018
P3	P3	0.62 ± 0.62	0.41 ± 0.46	-0.267	0.017
Pz	P3	0.63 ± 0.62	0.41 ± 0.47	0.174	0.043
P4	P3	0.28 ± 0.54	0.29 ± 0.73	-1.872	0.027

Potentials (mean ± SD) recorded at nine electrodes by logo after the general promotion or carnival promotion poster in the time window of 120–180, 270–320, and 370–630 ms, and the results of a two-sample paired *t*-test in two conditions on different sites.

For P2, the promotion condition showed a significant main effect [*F*(1,32) = 40.286, *p* < 0.001]. The mean amplitudes evoked by the e-commerce logo after the presentation of the carnival promotion posters were larger than the mean amplitudes evoked by the e-commerce logo after the presentation of the general promotion posters. A significant main effect was found for the distribution [*F*(8,256) = 52.409, *p* < 0.001]. The mean amplitudes distributed among the middle line were larger than the others. A significant main effect was also found for the promotion × distribution [*F*(8,256) = 46.451, *p* < 0.001]. Combining raw waveforms and scalp topographical mapping with variance analysis, a more positive P2 was found to have been elicited after the presentation of carnival promotion posters than general promotion, which was mainly distributed among the frontal-central scalp and was maximal at the center of the frontal-central scalp.

For N2, a significant main effect was found for the promotion condition [*F*(1,32) = 15.813, *p* < 0.001]. The mean amplitudes evoked by the e-commerce platform logo after the presentation of carnival promotion posters were smaller than that after the presentation of general promotion posters. A significant main effect was found for the distribution [*F*(8,256) = 109.628, *p* < 0.001], and the mean amplitudes distributed among the middle line were smaller than those of the others. Moreover, a significant main effect was found for the promotion × distribution interaction [*F*(8,256) = 9.645, *p* = 0.002]. A more negative N2 was evoked by the e-commerce platform logo after carnival promotion, which was also mainly distributed among the frontal-central scalp areas.

However, for P3, no significant main effect was found for the promotion status [*F*(1,32) = 0.012, *p* = 0.914], nor was there a significant main effect for the promotion condition × distribution interaction [*F*(8,256) = 2.860, *p* = 0.071]. Only the distribution indicated a significant main effect [*F*(8,256) = 9.357, *p* = 0.004]. Combining raw waveforms with scalp topographical mapping indicated that component P3 was mainly distributed among the central-parietal area and enhanced on the parietal area.

## 4. Discussion

In this study, we attempted to explore consumers’ cognitive differences when processing an unknown e-commerce brand after the presentation of two different online shopping promotion activities. The ERP components P2 and N2 evoked by the unknown e-commerce platform brand were stronger when the logos were processed after the presentation of the carnival promotion posters than the general promotion posters; however, this was not the case for the ERP component P3.

The P2 component, with an onset at about 150–200 ms after the stimulus appeared and which responded exclusively to the task relevance of the stimuli (Debora et al., 2009), is usually called the anterior P2 (P2a) (Geoffrey et al., 1996; Erika et al., 2013). Scholars have adopted several explanations for the cognitive process reflected by P2a. One interpretation is that P2a, mainly distributed among the frontal-central areas, reflects the target identification process (Potts, 2004; Erika et al., 2013). As mentioned in the experimental design, the unknown e-commerce platform brand logo stimuli were set as standard stimuli in sequences B and D, and subjects were asked to identify the target stimuli (i.e., the cartoon pictures) in these sequences. Before conducting sequences B or D, carnival promotion or general promotion posters had been presented in sequences A or C, respectively. Since the discount magnitude of the carnival promotion was designed to exceed the discount magnitude of the general promotion, subjects evaluated the carnival promotion stimuli as relevant even when they were not designated as the target (Geoffrey and Don, 2001; Potts, 2004). After the presentation of the carnival promotion posters, the larger P2 evoked by the unknown e-commerce brand logo indicated that preferential processing and more attention had been assigned to the unknown e-commerce brand logo compared to the general promotion posters. This explanation may suggest that the carnival poster could leave a more profound impression on subjects even when the sponsor of the carnival promotion is an unknown e-commerce brand, which is consistent with our general intuition. Another explanation is that the target sensitivity of P2a may contribute to the top-down facilitation of object recognition (Kopp et al., 2007), and P2a may reflect target sensitivity during visual object recognition. The e-commerce platform brand logo was visible at a fixed position (i.e., the upper right corner) in sequences A and C. During the experiments, a partially analyzed version of the visual input was projected from the visual areas to the prefrontal cortex (Victor and Pieter, 2000). Compared with the information carried by the general promotion posters, subjects may be more sensitive to the information carried by carnival promotion posters because this would enable them to save more money. Thus, the ERP P2 evoked by the e-commerce platform brand logo stimuli increased after the carnival promotion posters were presented.

In this study, promotion posters with the logo as the standard target were presented to subjects first in sequences A and C. The subjects were passively exposed to promotion information without requiring them to deliberately focus their attention on the logos in the posters at this stage. However, the results demonstrated that the e-commerce brand logo elicited a larger N2 after the presentation of carnival promotion posters than after the general promotion posters. It is generally accepted that N2 distributed in the front-central area of the scalp reflects the process of cognitive control (Eiichi and Yukihiko, 1992; Seiki et al., 1998). In this context, recent studies have shown that N2 seems to be related to the modulation mechanism of the orientation process of visual attention when a new stimulus is detected (Jonathan and Cyma, 2008; Friederike and Martin, 2014). This interpretation suggests that, even though participants were not aware of the logo in the promotion posters, nor did they consciously retrieve the logo, their implicit memory still affected their neural response to the logo stimuli in sequences B and D (Daniel, 1987). In the first phase of the experimental task, due to the different promotion magnitudes in the carnival and general posters, the logo information in the posters established implicit memory to different degrees in the subjects’ brains (Larry et al., 1987; Bruce and Abhijit, 2002). The implicit memory produced by the carnival promotion was stronger than the implicit memory produced by the general promotion. This speculation is consistent with prior studies that have claimed that implicit memory could be affected (Arnel, 1998; Kathmann et al., 2006). During the second phase of the experiment, while subjects counted the cartoon pictures, the different strengths of implicit memories led to a variation in the cognitive control process in response to logo stimulation. In sequences B and D, the subjects had to suppress the impact of the low-priority task target stimuli (logos) that were not related to the current task mission (identifying cartoon pictures). The subjects utilized more cognitive resources to suppress the implicit memory of the logo stimuli to maintain the high priority for the current task mission. As a result, a more negative N2 was evoked by the logos after the carnival promotion than after the general promotion. Another possible interpretation is that N2 may reflect the attention process, such as the detection of novel stimuli and the orienting of visual attention in the visual cortex. However, the component N2 under that interpretation was mainly distributed at the posterior scalp areas (Loveless, 1986; Salil and Pierre, 2005; Papaliagkas et al., 2011), which differed from what was found in our study.

Prior studies have reported that the positive potential P3, which is mainly distributed among parietal-central areas, may be caused by the classification process of a specific type of stimulus (Frederick et al., 2002; Qingguo et al., 2008; Ou et al., 2012). Accordingly, the evoked potential P3 in the 370–630 ms time window may reflect the process of classification of the brand. However, the results showed no significant differences between the mean amplitudes evoked by the e-commerce brand after the carnival promotion vs. after the general promotion. The reason for this may be that the e-commerce brand selected in this experiment was not well-known (as mentioned in section “Introduction”); the subjects may still regard it as an unknown brand. Different promotion posters may impose different initial impressions on the subjects, which could be reflected in the P2 and N2 components. However, if a further classification process were involved (e.g., whether to proceed with further actions based on the promotion activities), the results suggest that subjects may still require more information to arrive at a clear classification; thus, the evoked potential P3 did not show a significant difference.

The main differences between this study and prior studies are as follows. Prior studies related to online shopping carnival promotions have mainly focused on the topic of online shopping promotion, such as promotion strategies, firm performance, online and offline channel searching models, and the promotion’s effects on consumer behavior in terms of consumer perceptions of low prices, purchase intention, and decision-making. As can be seen from the previous literature mentioned in section “Introduction” (Svatosova, 2020; Li et al., 2022; Pan et al., 2022), scholars have conducted in-depth research on consumers’ purchasing behaviors in the context of online shopping, and their research perspectives have also undergone a transition from promotion strategies to consumer behaviors. The topics of this research have mainly focused on price and promotional incentives, consumer characteristics, situational factors, etc. The methodology of these studies was still observational investigation, causal models, experimental research, and data analysis. It can be truthfully said that the above research has achieved fruitful results. However, consumers’ behaviors and decision-making are controlled and directed by the brain. The research described above did not address how the brain integrates various pieces of promotional information to control and manage consumer behavior. In this study, ERP technology was used to directly and non-invasively observe the cognitive processes occurring when consumers received and processed different promotion information, which helps in revealing the cognitive mechanisms behind consumer behaviors. It can be seen from this study’s experimental results that consumers’ early cognitive processes showed significant differences after receiving general promotion stimulus information vs. carnival promotion stimulus information, even in an implicit paradigm. Combined with the cognitive meaning of the ERP components, we can know that after priming with carnival promotion posters, the unknown e-commerce platform stimuli elicited larger P2 and N2 components than did general promotion posters, indicating that the study of how online promotion stimulus information affects consumer behavior can be observed and explained by neuroimaging methods relatively accurately and objectively. In-depth and extensive consumer research using ERPs and other neuroimaging tools will help to strengthen the classical theory of consumer behavior and even make it possible to dynamically track the decision-making processes of consumers, providing fine, multidimensional data for research that can be used to predict consumer behavior more accurately. In the future, the behavior of a large population or a market could be predicted even by observing the cognitive activities of fewer subjects in the laboratory, which is of relatively significant importance.

## 5. Conclusion

In summary, this study attempts to explore differences in subjects’ subconscious cognitive process in response to an unknown e-commerce platform after viewing either online general promotion or carnival promotion content in an implicit task. The results showed that, after priming with different promotion activities, the early cognitive processes evoked in response to the e-commerce platform were reflected in the P2 and N2 components. The changes in P2 and N2 may reflect variations in target identification and the cognitive control mechanism processes evoked by the unknown e-commerce platform, which were affected by the implicit memory of the different promotion activities. A larger P2 and N2 were evoked by the unknown e-commerce platform after the presentation of the carnival promotion; however, further classification processes may not change based solely on promotion information. The results suggest that ERP components could be employed as indices to estimate the effectiveness of promotion methods even when the online shopping brand is totally unfamiliar to the subjects.

## 6. Contributions and implications

### 6.1. Theoretical contributions

Our research contributes to the literature in several ways. First, we are among the first to explore the effects of online promotion on consumers’ cognition in the context of an unknown platform through comparison of responses to a carnival promotion vs. a general promotion. Prior research has generally focused on well-known carnival promotions on established retailing platforms, where the effect of carnival promotion is mixed with that of the reputation of the established platform. Our work enriches the literature on carnival promotion by providing a positive link between carnival promotion and consumer cognition in the context of an unknown platform.

Second, our research sheds more light on the linkage between carnival promotion and consumer cognition. Compared to prior research that has focused on the effect of online promotion on consumers’ behavior, our study contributes to the literature by focusing on the early stage of consumers’ cognition as evoked by an online carnival promotion. Our findings provide a better understanding of whether there is variance in consumers’ attention and cognition control processes when they encounter carnival promotions vs. general promotions.

Third, our work uses the methods of neuroscience to estimate the effectiveness of promotion methods even when the online retailing platform is completely unfamiliar to the subjects. The findings also suggest that ERP components could be employed as a sensitive indicator for purposes of such an estimation and for investigating the cognitive processes of consumers as evoked by general promotions or carnival promotions.

### 6.2. Practical implications

Our findings also provide several important practical implications. First, a better understanding of promotion methods’ impact on consumer cognitive processes may help to improve the efficiency and accuracy of marketing strategies, especially for new firms that want to participate in the online retail business. With the help of neuroimaging tools such as ERPs, it can be expected that promotion strategists may be able to get a more accurate prediction of how effectively a promotion method of a given magnitude will attract the attention of consumers and even obtain a balance between costs and benefits.

Second, the study used an online shopping carnival held by a small, unknown e-commerce platform in order to exclude the influence of the reputation of a well-known e-commerce platform. Even so, the results found that subjects’ early cognitive processes showed significant differences after receiving information about an online shopping carnival promotion, as compared to after receiving information about a general promotion. Therefore, if companies want to effectively attract consumers’ attention, artificial online shopping carnivals seem to be a good idea. Following the examples of the *DOUBLE ELEVEN* or *Cyber Monday* carnivals that have achieved great market success, e-commerce platforms that have a dominant position in other markets could organize similar online shopping carnival activities. E-commerce platforms and consumers will both benefit from such action.

## 7. Limitations and future research

This research has some limitations. As mentioned in section “Introduction,” an online shopping carnival is different from a general promotion in its form and other aspects. In this study, the poster images were designed in a laboratory environment to simulate the excitement of carnival promotions, and the designed carnival posters mainly consisted of different discount formats, discount magnitudes, and limited-time promotions. Thus, it could not perfectly replicate the frenzied shopping atmosphere of a real-life carnival promotion; in addition, features unique to online carnival promotions, such as shopping games, live streaming shows, pleasure shared with others, and sharing links to get bigger discounts *via* SNS were not explored in this study.

Future research can focus on the following points. First, a more ingenious experimental design could be adopted to simulate real carnival promotion scenarios as much as possible in order to explore the consumer’s cognitive processes more accurately. Second, the scope of the research could be extended to offline consumption scenarios. In addition to the daily necessities which can be purchased online, it would also be interesting to explore whether services, high-value durable goods, or luxury goods could successfully adopt similar carnival promotion strategies. Third, future studies need to further consider the technical characteristics of neuroimaging tools, for example, by incorporating fMRI or eye-tracking technology and other physiological measurement technologies to explore the cognitive mechanisms at work in the process of consumer behavior.

## Data availability statement

The raw data supporting the conclusions of this article will be made available by the authors, without undue reservation.

## Ethics statement

The studies involving human participants were reviewed and approved by the Southwest University of Political Science and Law (SWUPL) Medical Ethics Committee. The patients/participants provided their written informed consent to participate in this study.

## Author contributions

WH designed the experiment. XZ executed and analyzed the data and wrote the manuscript with edits from WH. Both authors read and approved the final manuscript.
